# Prophylactic abdominal drainage following appendectomy for complicated appendicitis: A meta-analysis

**DOI:** 10.3389/fsurg.2022.1086877

**Published:** 2023-01-18

**Authors:** Jiankun Liao, Jiansheng Zhou, Jialei Wang, Guisheng Xie, Haotang Wei

**Affiliations:** Department of Gastrointestinal Surgery, The Second Nanning People's Hospital, The Third Affiliated Hospital of Guangxi Medical University, Nanning, China

**Keywords:** complicated appendicitis, appendectomy, abdominal drainage, metaanalysis, postoperative complications

## Abstract

**Background:**

To date, the value of prophylactic abdominal drainage (AD) following appendectomy in patients with complicated appendicitis (CA), including adults and children, has yet to be determined. This paper presents a meta-analysis of the effects of prophylactic AD on postoperative complications in patients with CA, with the goal of exploring the safety and effectiveness of prophylactic AD.

**Methods:**

PubMed, Science Direct, Web of Science, Cochrane Library, and Embase databases were searched for relevant articles published before August 1, 2022. The primary outcomes were the complication rates [overall incidence of postoperative complications, incidence of intra-abdominal abscess (IAA), wound infection (WI), and postoperative ileus (PI), and the secondary outcome was the perioperative outcome]. The meta-analysis was performed with STATA V. 16.0A.

**Results:**

A total of 2,627 articles were retrieved and 15 high-quality articles were eventually included after screening, resulting in a total of 5,123 patients, of whom 1,796 received AD and 3,327 did not. The results of this meta-analysis showed that compared with patients in the non-drainage group, patients in the drainage group had longer postoperative length of hospitalization (LOH) (SMD = 0.68, 95% CI: 0.01–1.35, *P* = 0.046), higher overall incidence of postoperative complications (OR = 0.50, 95% CI: 0.19–0.81, *P* = 0.01), higher incidence of WI (OR = 0.30, 95% CI: 0.08–0.51, *P* = 0.01) and PI (OR = 1.05, 95% CI: 0.57–1.54, *P* = 0.01), the differences were statistically significant. However, there was no significant difference in the incidence of IAA (OR = 0.10, 95% CI: −0.10 to 0.31, *P* = 0.31) between the two groups. The results of subgroup meta-analysis showed that in the adult subgroup, the overall incidence of postoperative complications in the drainage group was higher than that in the non-drainage group (OR = 0.67, 95% CI: 0.37–0.96, *P* = 0.01). However, there were no significant differences in IAA (OR = 0.18, 95% CI: −0.28 to 0.64, *P* = 0.45) and WI (OR = 0.13, 95% CI: (−0.40 to 0.66, *P* = 0.63) and PI (OR = 2.71, 95% CI: −0.29 to 5.71, *P* = 0.08). In the children subgroup, there were no significant differences in the incidence of IAA (OR = 0.51, 95% CI: −0.06 to 1.09, *P* = 0.08) between the two groups. The overall incidence of postoperative complications (OR = 0.46, 95% CI: 0.02–0.90, *P* = 0.04), incidences of WI (OR = 0.43, 95% CI: 0.14–0.71, *P* = 0.01) and PI (OR = 0.75, 95% CI: 0.10–1.39, *P* = 0.02) were significantly higher than those in the non-drainage group.

**Conclusion:**

This meta-analysis concluded that prophylactic AD did not benefit from appendectomy, but increased the incidence of related complications, especially in children with CA. Thus, there is insufficient evidence to support the routine use of prophylactic AD following appendectomy.

## 1. Introduction

Acute appendicitis is the most common cause of abdominal pain and one of the most common emergency cases in general surgery. It can be divided into acute non-complicated appendicitis and complicated appendicitis (CA) (including perforated or gangrenous appendicitis). According to statistics, about 58–89 persons per 100,000 will develop acute appendicitis, among which more than 90% of patients choose emergency appendectomy, including laparoscopic appendectomy (LA) and open appendectomy (OA) (1, 2). In addition, infection-related complications [e.g., intra-abdominal abscess (IAA), wound infection (WI), postoperative ileus (PI), etc.] occur in approximately 7%–10% of patients after appendectomy.

CA, defined as perforated or gangrenous appendicitis with or without abdominal abscess, is more prone to complications such as peritoneal infection or abscess following appendectomy (3). Some surgeons recommend prophylactic abdominal drainage (AD) after appendectomy to reduce the incidence of infectious complications after surgery. They believed that AD could reduce postoperative complications, especially the occurrence of IAA. However, more scholars have raised objections to this, believing that AD can not reduce the incidence of postoperative complications, but increase the incidence of related complications. Recently, a systematic review of six randomized controlled studies involving 521 patients with CA undergoing emergency appendectomy by Li and Cheng et al. found no significant difference in complication rates between the drainage and non-drainage groups (4–6). The role of prophylactic AD in reducing complications following OA in patients with CA is uncertain.

At present, the use of prophylactic AD after appendectomy still largely depends on the surgeon's preference and experience, and on the degree of appendicitis—non-complicated or complicated (7). The value of prophylactic AD for postoperative complications (such as IAA, WI, or PI) in patients with CA is uncertain and warrants further study.

Therefore, the purpose of this paper is to explore the effectiveness and safety of prophylactic AD in reducing the incidence of complications (IAA, WI, PI, etc.) after appendectomy (either open or laparoscopic) for CA in children/adults. The primary outcome of this meta-analysis is the incidence of complications (overall incidence of postoperative complications, incidence of IAA, WI, and PI). Secondary outcomes included surgical outcomes of interest [surgical time, time to resume a soft diet, and postoperative length of hospitalization (LOH)], rates of readmission within 30 days, and mortality.

## 2. Materials and methods

### 2.1. Literature search strategy

To analyze the effect of prophylactic AD on complications following appendectomy for CA, we conducted a systematic online literature search of the following databases: PubMed, Science Direct, Web of Science, Cochrane Library and Embase for relevant articles published before August 1, 2022. The search form of PubMed was: (((“Appendectomy” [Mesh]) OR (Appendectomy [Title/Abstract])) OR (Appendectomies [Title/Abstract])) AND (((“Drainage” [Mesh]) OR (Drainage [Title/Abstract])) OR (abdominal drainage [Title/Abstract])). In order to avoid omissions, a manual search was conducted for some relevant studies, among which all literature published in English was eligible for inclusion.

Due to the anonymity of the data, the requirement of informed consent was waived in this study.

### 2.2. Study selection

Two independent researchers (JL, JZ) respectively conducted literature search in the above databases, screened the literature according to the inclusion and exclusion criteria of the study, and included high-quality literature into the study by reading the full text. If any differences of opinion were encountered during the selection and inclusion of the literature, they were resolved by the third researcher (HW).

Inclusion criteria: (1) Studies were divided into drainage group and non-drainage group to compare whether or not prophylactic AD was performed after appendectomy for CA (either adults or children). The study had to report the incidence of postoperative complications (IAA, WI, PI or one of them); (2) The results of relevant research data can be directly or indirectly extracted from the research.

Exclusion criteria: (1) Patients without performing appendectomy and preventive AD were excluded; (2) Excluded case report, review, Conference Abstract, clinical answers and Letter; (3) If two studies were published by the same institution, the one with a smaller sample size was excluded; (4) Duplication, data loss and low-quality studies were excluded.

### 2.3. Data extraction

Two independent investigators (JL, JZ) pre-designed the data extraction table according to the purpose of the study, and subsequently independently reviewed and extracted the available data for each study, analyzed and compared the data. Extracted from into the research of data including research characteristics (such as the first author, year, the country, the data of accrual, research design, population and follow-up time), sample demographics [such as the number of patients, age, gender, body mass index (BMI), grade of appendicitis (8, 9)], surgical time, postoperative recovery (time to resume a soft diet, postoperative LOH), postoperative complications (IAA, WI, PI, etc.), rates of readmission within 30 days, and mortality. The corresponding author of the study will be contacted for additional data, if requested.

IAA formation was defined as abdominal abscess formation observed on ultrasound or CT within 30 days after surgery. WI was defined as clinical pus formation or erythema changes in the wound that required antibiotic treatment within 30 days after surgery. Moreover, PI was defined as symptoms and signs of abdominal distension, nausea, or vomiting, which were subsequently confirmed within 30 days after surgery based on continuous abdominal radiography, such as x-rays or CT.

### 2.4. Quality assessment of the studies

This meta-analysis was conducted on the recommendation of guidelines of the Preferred Reporting Items for Systematic Review and Meta-Analysis (PRISMA) 2020 Checklist. In this meta-analysis, the Newcastle-Ottawa Quality Assessment Scale (10) was used to evaluate the quality of retrospective case-control studies (NOS, 9 points) and cohort studies (NOS, 13 points). It describes the population selection, comparability, and exposure assessment or outcome assessment for each study. In addition, the Jadad score (5 points) was used to evaluate the quality of randomized clinical trials (RCTs) (11). Its procedures included randomization, double blinding, dropout, and loss to follow-up. A NOS score ≥6 or a Jadad score >3 indicates a high-quality score. All articles included in our meta-analysis were of high quality.

### 2.5. Statistical analysis

This meta-analysis was performed using STATA V. 16.0A (STATA Corp, College Station, TX, US). Odds Ratio (OR) and Standard Mean Difference (SMD) with 95% Confidence Interval (CI) were used to evaluate dichotomous and continuous data. The Cochrane chi-square test was used to evaluate heterogeneity among studies. When *I*^2^ < 50% and statistical heterogeneity was small, the fixed effects model was used for data analysis; for everyone else, the random effects model was used. Sensitivity analysis and Egger's test were used to detect research bias. And *P* < 0.05 was defined as statistically significant.

## 3. Results

### 3.1. Search results

**Figure 1:**
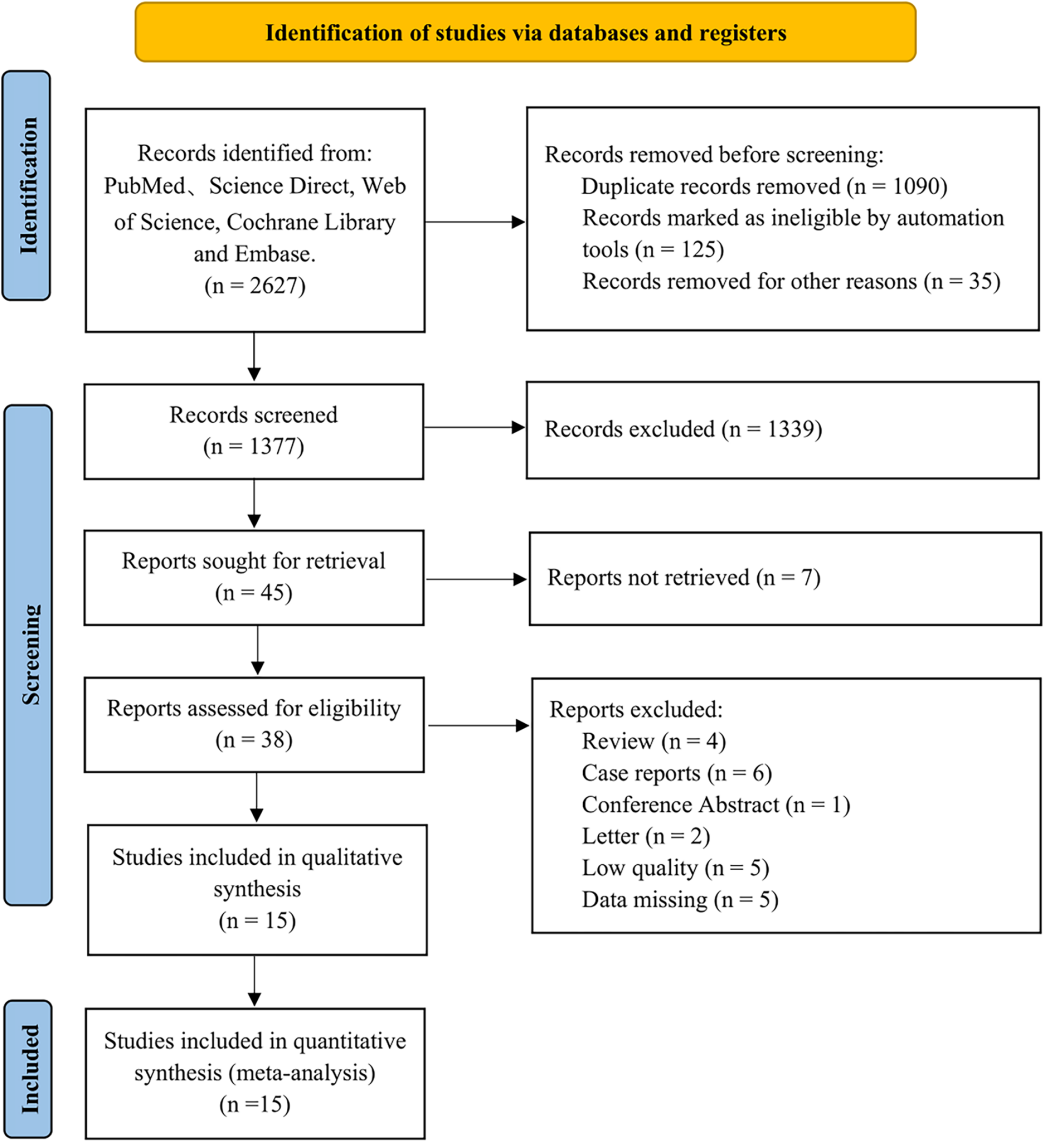
Flow diagram of literature screening.

A total of 2,627 studies were identified after an initial search based on the search strategy. Among them, 779 articles in PubMed, 628 articles in ScienceDirect, 438 articles in the Web of Science, 79 articles in the Cochrane Library, 700 articles in EMBASE, and 3 articles in manual search. Subsequently, 1,250 articles were removed due to duplications and ineligibility; 1,339 articles were excluded by carefully reviewing titles and/or abstracts. Then, 45 articles were evaluated through full text, and 30 articles were excluded. Finally, 15 articles with 5,123 patients published up to August 1, 2022 were included (8, 12–25). The study selection flow diagram is shown in Figure 1.

### 3.2. Sensitivity analysis and publication bias

**Figure 2:**
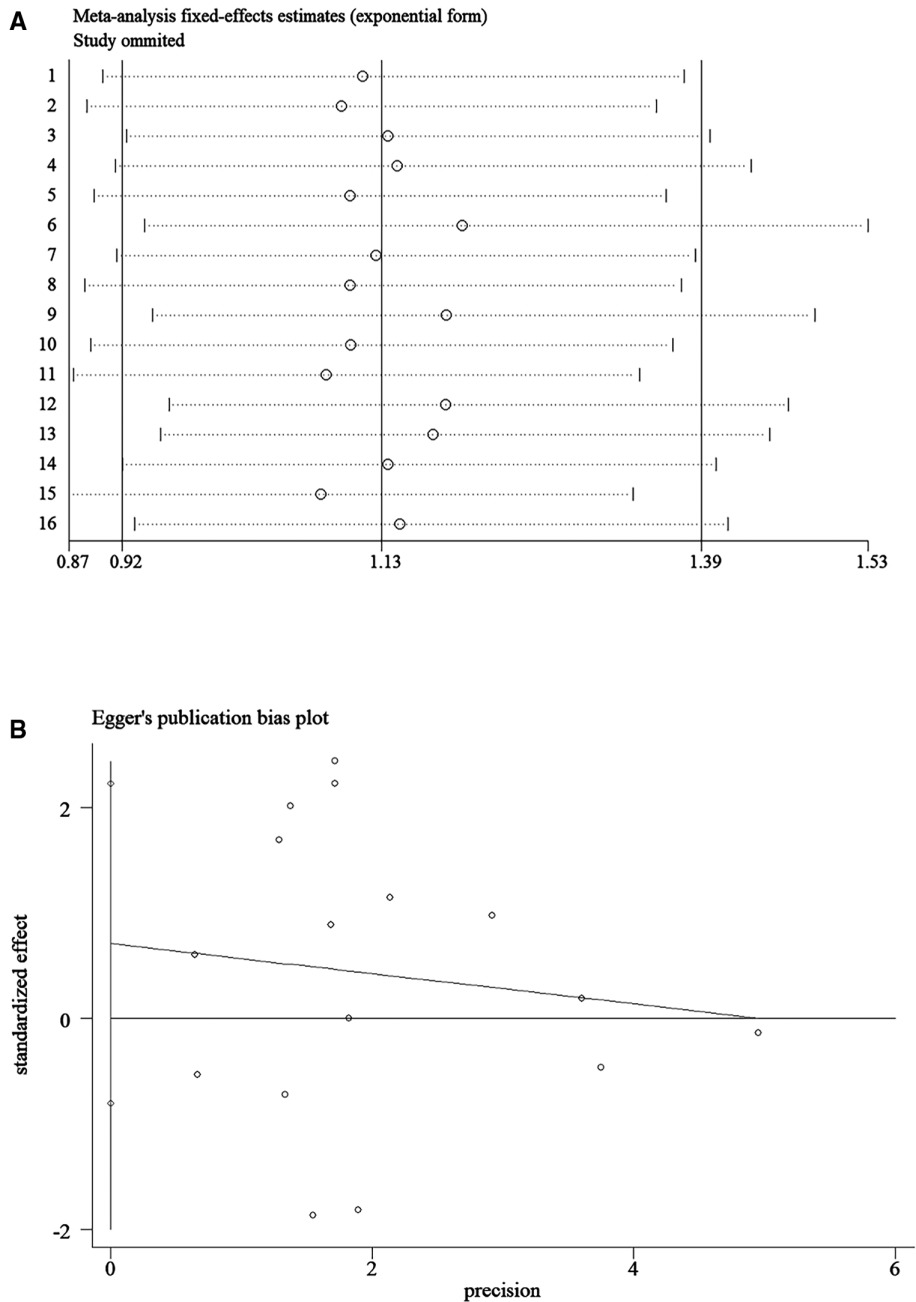
Sensitivity analysis and Egger's publication bias plot of the incidence of intra-abdominal abscess, (**A**) sensitivity analysis; (**B**) Egger's publication bias plot.

In this meta-analysis, sensitivity analysis was performed by excluding studies one by one to estimate the overall impact of each study, and the results showed little change, indicating that the results of meta-analysis had strong stability (Figure 2A presents IAA as an example). We also performed publication bias analysis on the outcome results, and the Egger' test showed no publication bias (Figure 2B presents IAA as an example, *P* = 0.332).

### 3.3. Study characteristics

A total of 15 studies with 5,123 patients from 12 countries were included in this meta-analysis, including 1 randomized controlled trial (25), 3 retrospective cohort studies (8, 16, 18) and 11 retrospective case-control studies (12–15, 17, 19–24). Sample size of each study varied, ranging from 16 to 458 patients. In particular, one article compared and analyzed the effects of two different types of drainage on postoperative complications (12). In addition, 4 articles only included CA in adult (13, 14, 16, 23), while 6 articles only included CA in children (12, 15, 17, 20, 24, 25), and the others included both adults and children (8, 18, 19, 21, 22). All articles included in this meta-analysis are of high quality after quality evaluation. Table 1 presents the basic characteristics and quality evaluation results of included studies.

**Table 1:** The basic characteristics and quality evaluation results of included studies.

Study	Year	Country	Dates of accrual	Study design	Cases/drain: no drain	Population	Follow-up (months)	NOS stars	Jadad score
Liao	2022	Tai wan	2014.01–2018.12	RCS	192: 229	CA	1	10★	-
Tsai (PD/ND)	2021	Taiwan	2012.01–2018.11	RCC	19: 86	CA in children	24	7★	-
Tsai (JP/ND)	2021	Taiwan	2012.01–2018.11	RCC	16: 86	CA in children	24	7★	-
Qian	2021	USA	2017.01–2018.06	RCC	159: 475	CA in adults	1	8★	-
Nazarian	2021	UK	2018.03–2018.11	RCC	26: 50	CA in adults	1	6★	-
Fujishiro	2021	Japan	2015.01–2015.12	RCC	458: 1,304	CA in children	1	8★	-
Miranda-Rosales	2019	Peru	2014.01–2014.12	RCS	100: 50	CA in adults	NA	9★	-
Aneiros castro	2018	Spain	2000.01–2013.12	RCC	117: 75	CA in children	NA	7★	-
Abdulhamid	2018	Iraq	2014.04–2017.06	RCS	114: 113	CA	NA	10★	-
Schlottmann	2016	Argentina	2005.01–2015.06	RCC	56: 169	CA	1	7★	-
Song	2015	Korea	2003.03–2012.09	RCC	108: 234	CA in children	NA	7★	-
Beek	2015	Netherlands	2011.01–2013.08	RCC	79: 120	CA	NA	7★	-
PAKULA	2014	USA	2007.01–2011.06	RCC	43: 105	CA	NA	7★	-
Allemann	2011	Switzerland	2003.11–2007.06	RCC	130: 130	CA in adults	12	7★	-
Narci	2007	Turkey	1999.01–2003.03	RCC	109:117	CA in children	12	6★	-
Tander	2003	Turkey	NA	RCT	70:70	CA in children	NA		4

NOS, Newcastle-Ottawa Quality Assessment Scale; RCS, retrospective cohort study; RCC, retrospective case-control study; RCT, randomized controlled trial; CA, complicated appendicitis; NA, not applicable.

### 3.4. Demographics of the patients

Not all studies reported sex ratio and BMI, and only two studies reported grade of appendicitis and performed an inter-group analysis (8, 13). Table 2 shows the demographic results for the patients of included studies.

**Table 2:** Demographic results of patients of included studies.

Study	No. of patients	Age (years)/drain: no drain	Gender (male/female)		BMI (kg/m^2^)	AAST grade (II/III/IV/V, *n*)	
			Drainage group	No drainage group		Group A	Group B
Liao	421	46.38 ± 19.01: 42.44 ± 19.93	125/67	132/97	24.08 ± 4.55: 23.57 ± 4.37	21/31/72/67	52/46/80/51
Tsai (PD/ND)	105	11.0 ± 3.92: 11.5 ± 3.59	13/6	56/30	NA	NA	NA
Tsai (JP/ND)	102	9.3 ± 3.64: 11.5 ± 3.59	9/7	56/30	NA	NA	NA
Qian	634	52 (39–62): 48 (33–61)	97/62	260/215	NA	-/62/46/51	-/249/122/104
Nazarian	76	39.62 (17–82): 37.42 (19–79)	NA	NA	NA	NA	NA
Fujishiro	1,762	NA	256/202	781/523	NA	NA	NA
Miranda-Rosales	150	35.00 (15–72): 36.76 (15–70)	60/40	30/20	NA	NA	NA
Aneiros castro	192	7.57 ± 3.5: 8.07 ± 3.2	73/44	48/27	NA	NA	NA
Abdulhamid	227	31.75: 30.77	54/60	60/53	NA	NA	NA
Schlottmann	225	43.3 (16–92): 43.1 (16–93)	36/20	98/71	NA	NA	NA
Song	342	9.92 ± 4.25: 10.97 ± 4.04	60/48	141/93	19.88 ± 4.81: 19.31 ± 4.16	NA	NA
Beek	199	37 (6–83): 33 (3–82)	44/35	63/57	NA	NA	NA
PAKULA	148	32 ± 14: 29 ± 10	33/10	80/25	NA	NA	NA
Allemann	260	38 (16–75): 31 (16–71)	72/58	83/47	24.2 (17.2–43.4): 24.5 (16.7–40.1)	NA	NA
Narci	226	8.7 ± 3.3: 8.5 ± 3.6	75/34	75/42	NA	NA	NA
Tander	140	6.89 ± 3.5: 7.31 ± 3.4	50/20	52/18	NA	NA	NA

BMI, body mass index; kg, kilogram; m, meter; AAST, American association for the surgery of trauma; NA, not applicable.

### 3.5. Surgical time

**Figure 3:**
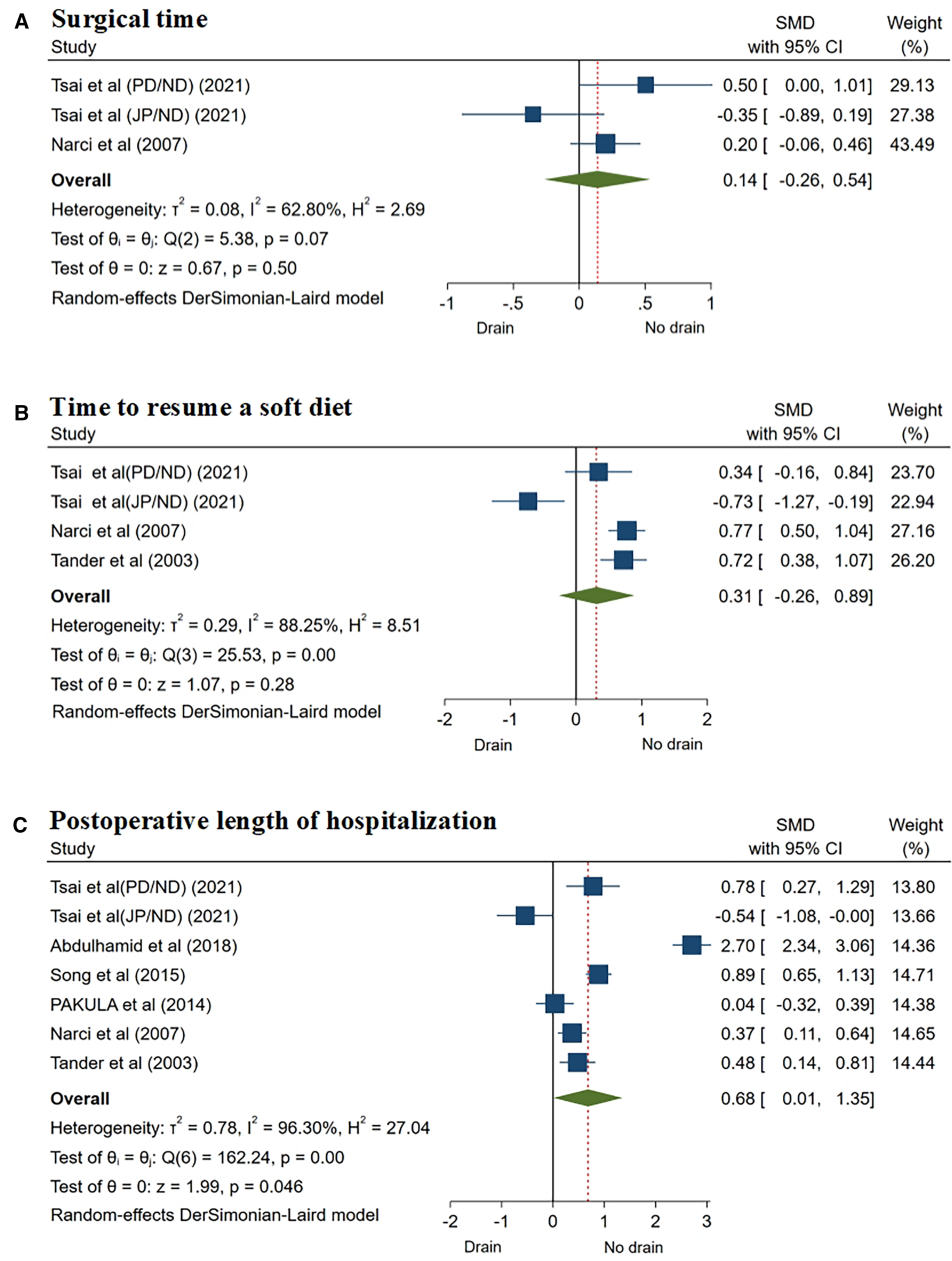
Forest plots of meta-analysis and subgroup meta-analysis comparing surgical feature and postoperative recovery, (**A**) surgical time; (**B**) time to resume a soft diet; (**C**) postoperative length of hospitalization.

Two studies (12, 24) reported the surgical time. Due to the heterogeneity of the reported results (*I*^2^ = 62.8%, *P* = 0.07), the random-effects model was selected, and there was no significant difference in surgical time between the two groups (SMD = 0.14, 95% CI: −0.26 to 0.54, *P* = 0.50) (Figure 3A; Table 3).

**Table 3:** Meta-analysis of surgical feature, postoperative complications, and postoperative recovery.

Variables	No. of studies	No. of patients (drain: no drain)	Heterogeneity		Model	SMD/OR (95% CI)	*P* value
			*I*^2^, %	*P* value			
Surgical time	2	144: 203	62.80	0.07	Random	0.14 (−0.26, 0.54)	0.50
Postoperative LOH	6	479: 725	96.30	0.01	Random	0.68 (0.01, 1.35)	0.046
Time to resume a soft diet	3	214: 273	88.25	0.01	Random	0.31 (−0.26, 0.89)	0.28
Overall incidence of postoperative complications	12	1,704: 2,535	71.08	0.01	Random	0.50 (0.19, 0.81)	0.01
IAA	15	1,796: 2,939	46.13	0.02	Fixed	0.10 (−0.10, 0.31)	0.31
WI	13	1,697: 2,665	0	0.65	Random	0.30 (0.08, 0.51)	0.01
PI	7	657: 861	0	0.46	Random	1.05 (0.57, 1.54)	0.01
Readmission within 30 days	4	888: 1,740	48.55	0.12	Random	−0.10 (−0.46, 0.27)	0.61

SMD, standard mean difference; OR, odds ratio; CI, confidence interval; LOH, length of hospitalization; IAA, intra-abdominal abscess; WI, wound infection; PI, postoperative ileus.

### 3.6. Postoperative recovery

#### 3.6.1. Time to resume a soft diet

Three studies (12, 24, 25) reported the time to resume a soft diet, using the random effect model (*I*^2^ = 88.25%, *P* = 0.01), and the results showed that there was no significant difference between the two groups (SMD = 0.31, 95% CI: −0.26 to 0.89, *P* = 0.28) (Figure 3B; Table 3).

#### 3.6.2. Postoperative length of hospitalization

The meta-analysis of 6 studies (12, 18, 20, 22, 24, 25), using the random effect model (*I*^2^ = 96.3%, *P* = 0.01), showed that the postoperative LOH of patients in the drainage group was significantly longer than that in the non-drainage group (SMD = 0.68, 95% CI: 0.01–1.35, *P* = 0.046) (Figure 3C; Table 3).

### 3.7. Postoperative complications

#### 3.7.1. Overall incidence of postoperative complications

**Figure 4:**
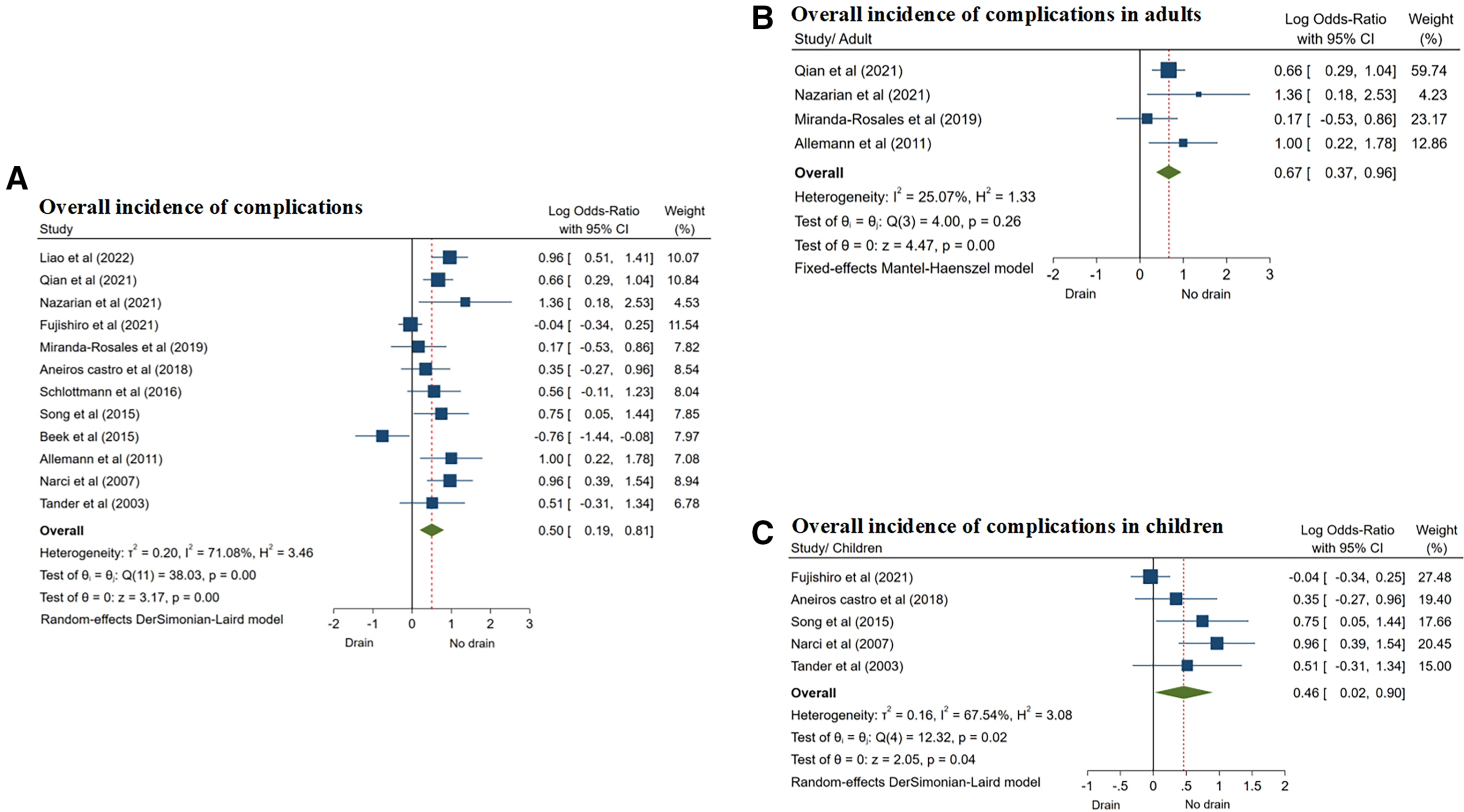
Forest plots of meta-analysis and subgroup meta-analysis comparing morbidity, (**A**) overall incidence of complications; (**B**) overall incidence of complications in adults; (**C**) overall incidence of complications in children.

Twelve studies (8, 13–17, 19–21, 23–25) reported the overall incidence of postoperative complications. Due to the heterogeneity of the results (*I*^2^ = 71.08%, *P* = 0.01), the random effect model was used in the meta-analysis. The results showed that the overall incidence of postoperative complications in the drainage group was higher than that in the non-drainage group, and the difference was statistically significant (OR = 0.50, 95% CI: 0.19–0.81, *P* = 0.01) (Figure 4A; Table 3).

According to the included population, the studies were divided into two subgroups: adults and children. Four studies (13, 14, 16, 23) only included CA in adults. The results showed that in the adult subgroup, the overall incidence of postoperative complications in the drainage group was also higher than that in the non-drainage group (OR = 0.67, 95% CI: 0.37–0.96, *P* = 0.01) (Figure 4B; Table 4), and the same results were also shown in 5 studies of CA in children subgroup (15, 17, 20, 24, 25) (OR = 0.46, 95% CI: 0.02–0.90, *P* = 0.04) (Figure 4C; Table 4), and the differences were statistically significant.

**Table 4:** Subgroup meta-analysis of postoperative complications.

Variables	No. of studies	No. of patients (drain: no drain)	Heterogeneity		Model	SMD/OR (95% CI)	*P* value
			*I*^2^, %	*P* value			
Overall incidence of postoperative complications							
Children	5	862: 1,312	67.54	0.02	Random	0.46 (0.02, 0.90)	0.04
Adults	4	415: 705	25.07	0.26	Fixed	0.67 (0.37, 0.96)	0.01
IAA							
Children	6	897: 1,498	55.91	0.03	Random	0.51 (−0.06, 1.09)	0.08
Adults	4	415: 705	0	0.44	Random	0.18 (−0.28, 0.64)	0.45
WI							
Children	6	897: 1,498	0	0.57	Random	0.43 (0.14, 0.71)	0.01
Adults	4	415: 705	20.36	0.29	Fixed	0.13 (−0.40, 0.66)	0.63
PI							
Children	5	439: 582	0	0.55	Random	0.75 (0.10, 1.39)	0.02
Adults	1	26: 50	NA	NA	Fixed	2.71 (−0.29, 5.71)	0.08

SMD, standard mean difference; OR, odds ratio; CI, confidence interval; IAA, intra-abdominal abscess; WI, wound infection; PI, postoperative ileus.

#### 3.7.2. Intra-abdominal abscess

**Figure 5:**
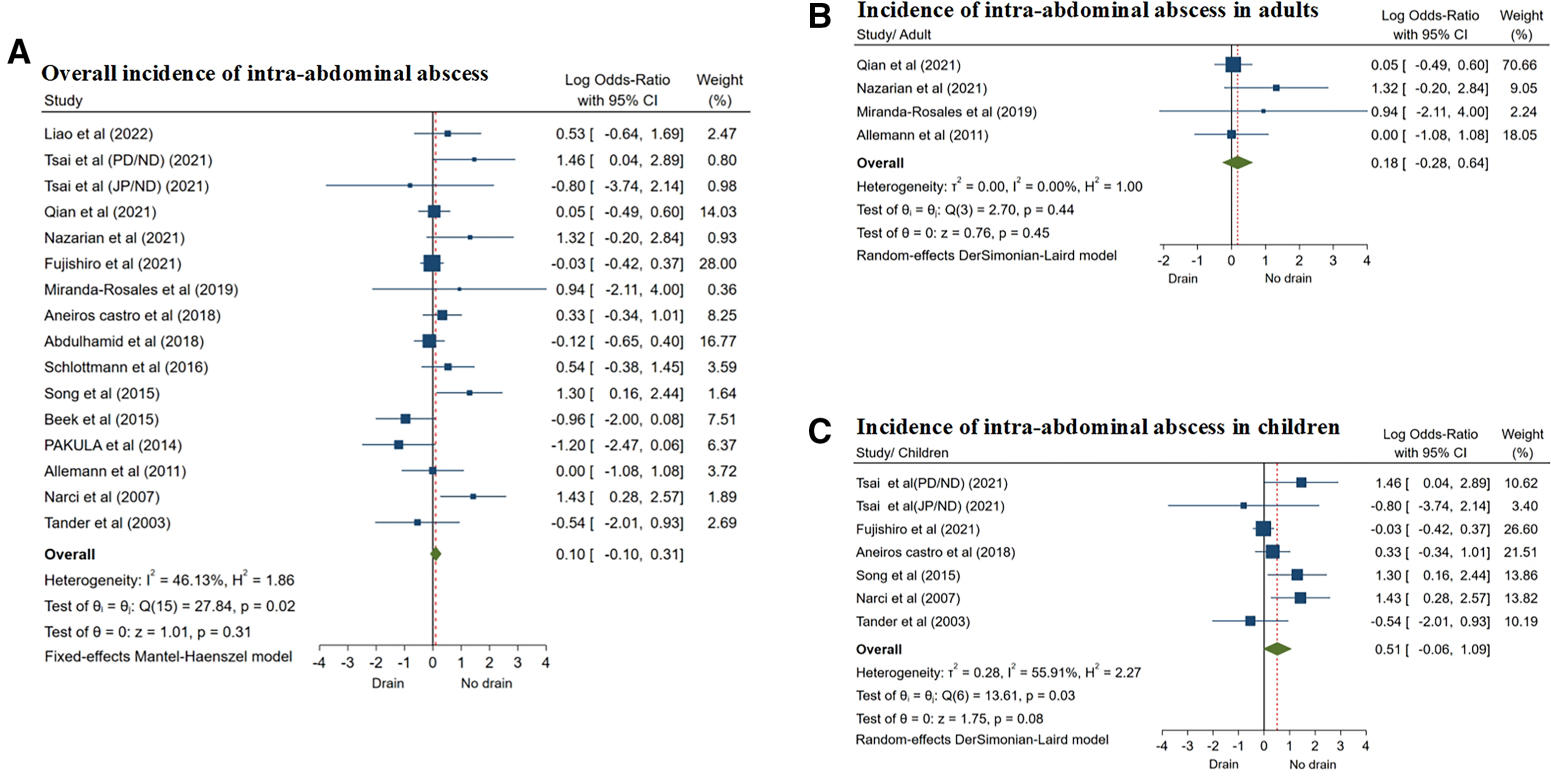
Forest plots of meta-analysis and subgroup meta-analysis comparing intra-abdominal abscess, (**A**) overall incidence of intra-abdominal abscess; (**B**) incidence of intra-abdominal abscess in adults; (**C**) incidence of intra-abdominal abscess in children.

All 15 studies (8, 12–25) reported the incidence of postoperative IAA. The fixed effects model was used in the meta-analysis (*I*^2^ = 46.13%, *P* = 0.02), and the results showed that there was no significant difference in the incidence of postoperative IAA between the two groups (OR = 0.10, 95% CI: −0.10 to 0.31, *P* = 0.31) (Figure 5A; Table 3).

Studies were also divided into adults and children's subgroups. A total of 4 studies (13, 14, 16, 23) included CA in only adults, and 6 studies (12, 15, 17, 20, 24, 25) reported CA in children. The results of subgroup analysis showed that there was no significant difference in the incidence of IAA between the two groups of patients in adults subgroup (OR = 0.18, 95% CI: −0.28 to 0.64, *P* = 0.45) (Figure 5B). The results were similar in the children subgroup (OR = 0.51, 95% CI: −0.06 to 1.09, *P* = 0.08) (Figure 5C; Table 4). Subgroup analyses were performed using a random effects model.

#### 3.7.3. Wound infection

**Figure 6:**
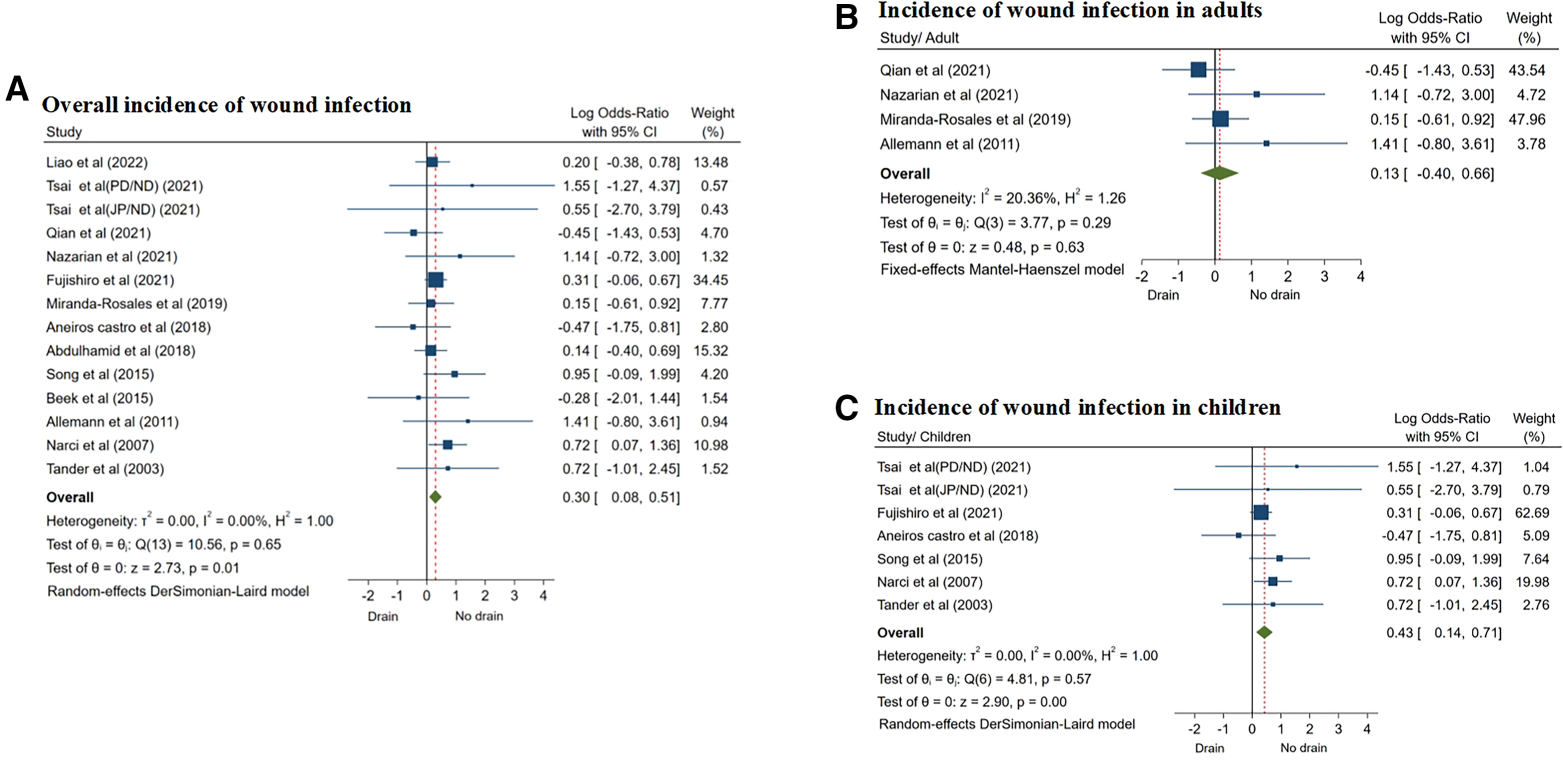
Forest plots of meta-analysis and subgroup meta-analysis comparing wound infection, (**A**) overall incidence of wound infection; (**B**) incidence of wound infection in adults; (**C**) incidence of wound infection in children.

Thirteen studies (8, 12–18, 20, 21, 23–25) reported the incidence of postoperative WI. According to the random effects model (*I*^2^ = 0.00%, *P* = 0.65), the incidence of WI was significantly higher in patients in the drainage group than in the non-drainage group (OR = 0.30, 95% CI: 0.08–0.51, *P* = 0.01) (Figure 6A; Table 3), and the difference was statistically significant.

Similarly, there were 4 studies (13, 14, 16, 23) including adults with CA and 6 studies (12, 15, 17, 20, 24, 25) including children. However, the results of subgroup analysis showed that there was no significant difference in the incidence of WI between the two groups of patients in adults (OR = 0.13, 95% CI: −0.40 to 0.66, *P* = 0.63) (Figure 6B). On the contrary, in the children subgroup, the incidence of WI in the drainage group was significantly higher than that in the non-drainage group (OR = 0.43, 95% CI: 0.14–0.71, *P* = 0.01) (Figure 6C; Table 4).

#### 3.7.4. Postoperative ileus

**Figure 7:**
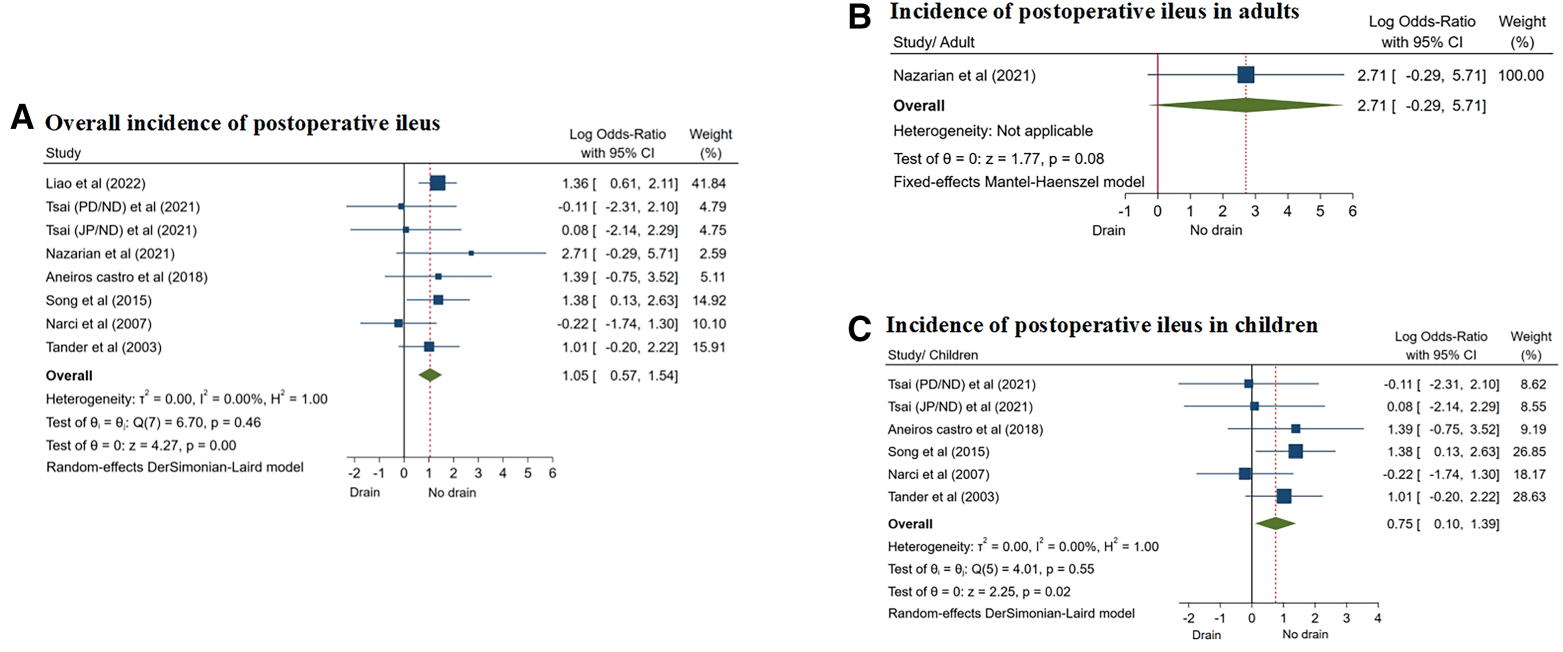
Forest plots of meta-analysis and subgroup meta-analysis comparing postoperative ileus, (**A**) overall incidence of postoperative ileus; (**B**) incidence of postoperative ileus in adults; (**C**) incidence of postoperative ileus in children.

The incidence of PI was reported in 7 studies (8, 12, 14, 17, 20, 24, 25), and the random effect model was used for analysis (*I*^2^ = 0.00%, *P* = 0.46). The results showed that the incidence of PI in the drainage group was significantly higher than that in the non-drainage group (OR = 1.05, 95% CI: 0.57–1.54, *P* = 0.01) (Figure 7A; Table 3).

They were divided into two subgroups based on the study population: adults and children. Only one study (14) reported CA in adults, and there was no significant difference in the incidence of PI between the two groups (OR = 2.71, 95% CI: −0.29 to 5.71, *P* = 0.08) (Figure 7B). However, there were 5 studies (12, 17, 20, 24, 25) on children with CA, and the results of subgroup analysis showed that the incidence of PI in the drainage group was significantly higher than that in the non-drainage group (OR = 0.75, 95% CI: 0.10–1.39, *P* = 0.02) (Figure 7C; Table 4).

### 3.8. Readmission and mortality

Four studies (8, 13, 15, 21) reported the rate of readmission within 30 days after surgery, using the fixed effects model (*I*^2^ = 48.55%, *P* = 0.12). The results showed that there was no significant difference in the readmission rate between the two groups (OR = −0.10, 95% CI: −0.46 to 0.27, *P* = 0.61) (Table 3).

A total of 4 studies (16, 18, 19, 22) reported mortality, and no perioperative deaths were reported in any of the 4 studies.

## 4. Discussion

It is well known that appendectomies, both laparoscopically assisted and laparotomy, are the primary treatment for CA, which has become a consensus. Due to the influence of inflammatory factors, appendectomies are often prone to complications, such as IAA, WI and PI. Clinically, AD is used prophylactically by many general surgeons to drain infectious effusions, with the desired goal of reducing the incidence of postoperative complications (26, 27). However, over the past 30 years, most studies (8, 14, 20, 23, 24) have shown that prophylactic AD does not reduce postoperative complications, but increases the incidence of related complications (WI, PI, etc.). Conversely, Pakula et al. (12, 21, 22) positively concluded that the use of drainage tubes reduced the risk of IAA formation, the overall incidence of postoperative complications, and the reintervention rate, and subsequently recommended prophylactic AD after appendectomy for CA.

To the best of our knowledge, only Li and Cheng et al. (4–6) have reviewed the use of AD for complications after OA. However, the authors themselves stated that the evidence from this systematic analysis was of low certainty and that there was no evidence that patients undergoing OA benefit from prophylactic AD. So far, the application value of prophylactic AD remains controversial and no final consensus has been reached. Therefore, we conducted this systematic meta-analysis including 15 high-quality studies. The results of meta-analysis showed that there were no statistically significant differences between the two groups in surgical time, time to resume a soft diet, IAA, and readmission within 30 days, but AD significantly increased the overall incidence of postoperative complications, and it increases the incidence of WI and PI, and prolongs the postoperative LOH. Moreover, no mortality was found in either group. However, it is interesting to note that the increased incidence of WI and PI occurred only in the children subgroup, and AD did not affect the incidence of WI and PI after CA in adults.

The meta-analysis showed that compared with the non-drainage group, the drainage group had no statistically significant differences in surgical time, time to resume a soft diet, and readmission rate within 30 days after surgery. Although this is not supported by Tsai and Narci et al. (12, 24). In addition, the results showed that the use of AD was associated with a longer postoperative LOH. Most of the included studies also support the idea that AD may prolong postoperative LOH (8, 12–15, 18–20). Allemann et al. considered that the AD tube would act as a foreign body, leading to an increased incidence of PI and longer time to resume a soft diet, so patients were later discharged (23). Furthermore, Schlottmann et al. (19) analyzed the reasons for the prolonged postoperative LOH, considering that it was not only related to physical factors, but also included the psychological activity factors of the patients. Because the patient seems to be taking on the role of being sick, he gets up and moves less until the drain is removed. However, a small number of studies (21, 22) have suggested that there was no significant difference in postoperative LOH between the two groups, and the placement of AD did not significantly increase postoperative LOH. There are even data from a single study showing that patients in the drainage group had a shorter postoperative LOH than those in the non-drainage group (12), perhaps because active drainage of the JP (Jackson-Pratt) drainage tube promotes the elimination of pus or suppurative ascites, which not only reduces the risk of IAA formation, but also shortens the postoperative LOH.

The results of the meta-analysis also showed statistically significant differences in the overall incidence of postoperative complications, WI and PI between the two groups, and the incidence of above complications in the drainage group was higher than that in the non-drainage group. Allemann et al. (23) suggested that the significantly higher incidence of postoperative complications in the drainage group was caused by a large number of abdominal wall infections associated with the drainage exit site. The presence of drainage tube not only causes contaminated fluid to flow into the subcutaneous tissue along the drainage tube, leading to WI (28, 29). In addition, the presence of drainage tube will also increase foreign body reaction, which can exacerbate bowel movements and lead to small bowel obstruction. This finding is consistent with the study of Pessaux et al. and the meta-analysis by Gurusamy et al. (30, 31). Nevertheless, Nazarian et al. found that preoperative c reactive protein (CRP) was significantly elevated in patients of CA with postoperative drainage. Moreover, a large number of studies have shown that preoperative CRP level is an effective predictor of WI after appendectomy (32, 33). As a result, it is not clear whether the severity of the disease or the presence of foreign body insertions is responsible for the formation of infection in patients who drain and develop IAA following surgery.

In addition, our meta-analysis showed that the incidence of postoperative IAA varied greatly between the two groups. The incidence of IAA in the drainage group was 0.0%–43.9%, while that in the patients without drainage tube insertion was 0.0%–46.9%. There was no significant difference between the two groups. This is consistent with the findings of Qian and Abdulhamid et al. (12–19, 21, 23, 25), which suggest that AD does not prevent the formation of IAA. Moreover, 3 studies (8, 20, 24) directly showed that prophylactic AD increased the incidence of IAA. These authors considered that the drainage tube may be bent and blocked by blood clots, pus, infected debris, fibrin, or other substances, resulting in drainage dysfunction; The tip of the AD tube may not drain the site of abscess formation, but the presence of the drainage tube can also increase foreign body reactions, impair natural immune defense mechanisms, and aggravate infection (24, 34). However, 2 studies (12, 22) have shown that JP drainage could reduce the incidence of IAA.

In most of the included studies, ordinary rubber tube or silicone tube was used for passive drainage following appendectomy, and the drainage effect was poor, which may be one of the reasons that prophylactic AD could not reduce postoperative complications and even increase the incidence of complications. A Japanese study (15) also showed no advantage of routine drainage in terms of postoperative outcomes or LOH in children with CA. In view of the influence of drainage tube types, Tsai et al. (12) proposed new drainage methods: using a JP drainage tube (Jackson-Pratt® flat perforated drainage tube, 7 mm diameter), compared with using a Penrose drainage tube (silicone Penrose drainage, 6 mm wide) or no drainage tube, the negative pressure created by the closed suction system in the JP drainage system allows drainage of pus or suppurative ascites, providing active drainage that can shorten postoperative LOH and reduce the risk of postoperative IAA. However, the small sample size and the limitations of the retrospective design precluded conclusions of high evidence.

The positive role of JP drainage is also supported by Pakula et al. (22, 23), who also reported the positive effects of active drainage using closed system peritoneal drainage tubes (e.g., JP drainage), including a reduced risk of postoperative IAA, as well as lower rates of re-intervention and readmission. However, these findings are not supported by Song et al. (20), whose data did not show an advantage of JP drainage, rather increased the incidence of postoperative complications, including IAA. But the article did not describe whether the drainage tube has a negative pressure attraction. Therefore, the positive effect of the type of drainage (JP drainage) on postoperative complications in patients with CA is not well established and further studies are needed.

To analyze the effect of AD in different populations, we divided the included studies into two subgroups: adults and children and conducted subgroup meta-analysis. The results showed that AD significantly increased the overall incidence of postoperative complications, but did not increase or decrease the incidence of IAA. The results were consistent in both adults and children. However, the incidence of WI and PI was significantly different only in the children subgroup and not in the adults subgroup. In other words, AD significantly increased the postoperative incidences of WI and PI for CA in children, but did not have a significant effect on the incidence of WI and PI for CA in adults. In this regard, Aneiros Castro et al. (17) argue that the higher rate of perforation in children with CA compared to adults, ranging from 20% to 76%, may lead to a significant increase in postoperative complications, from which prophylactic use of AD does not benefit (35).

The overall incidence of postoperative complications is another important consideration when comparing drainage with non-drainage. The difference in the overall incidence of postoperative complications between the two groups was statistically significant, possibly because the total complications included not only the major complications, such as IAA, WI, and PI, but also postoperative pain and pneumonia. Previous studies have shown that drainage tube placement can increase the rate of postoperative pain in patients (12). However, in the subgroup-analysis, there were no significant differences in the major complications of IAA, WI, and PI between the two groups in adults subgroup. In contrast, in the children subgroup, the use of prophylactic AD significantly increased the incidence of WI and PI. This suggests that adult patients with CA do not benefit from prophylactic AD, but do not have an increased incidence of postoperative complications. However, prophylactic AD increases the incidence of postoperative complications for CA in children, especially WI and PI.

It is worth considering that studies (8, 13, 36) have shown that the grade of appendicitis is an independent risk factor for postoperative IAA, and also a risk factor for postoperative LOH, so it has a certain impact on postoperative complications. Many studies did not consider the influence of severity grade of appendicitis on postoperative complications and failed to control the influence of its factors in the comparison of drainage tube placement or not. In some studies, patients in the drainage group were more likely to choose patients with a higher grade of appendicitis (24). For example, in the study of Aneiros Castro et al. (17), there was a significant difference in the incidence of preoperative peritonitis between the drainage group and the non-drainage group, and patients in the drainage group had higher grade of appendicitis. In addition, the application value of prophylactic AD in laparoscopic or open appendectomy remains to be determined, which needs to be further determined in subsequent studies.

Both strengths and limitations arise from this meta-analysis. The advantage of this meta-analysis is that the number of results comparing AD after appendectomy in CA is the largest and most comprehensive to date, which may be of reference. In addition, we divided into two subgroups and children to analyze the application value of AD in different age groups. However, there are also some limitations to this meta-analysis. First, among the 15 studies, 14 were clinical observational studies and only 1 was a randomized controlled trial. Secondly, only two subgroups, adults and children, were included in the meta-analysis, and two subgroups, laparoscopic and open surgery, were not included in the meta-analysis to analyze the impact of surgical methods. Finally, only a few studies included appendicitis grade in their baselines and failed to consider the effect of grade of appendicitis on postoperative complications. Patients with AD may have a higher grade of appendicitis, so they were not finely stratified. The advantages and disadvantages of AD in different grades of appendicitis remain to be determined.

## 5. Conclusion

The meta-analysis of 15 studies showed that prophylactic AD did not benefit from appendectomy in adults with CA, but it did not increase the incidence of postoperative complications, although it could prolong the postoperative LOH. However, the prophylactic use of AD in children will increase the incidence of postoperative complications, especially WI and PI. However, due to the limitations of the study design, the effect of the grade of appendicitis on postoperative complications and the role of AD were not analyzed stratified.

In conclusion, the results of our meta-analysis do not encourage the routine prophylactic use of AD after appendectomy in adults, but the prophylactic use of AD can be considered cautiously according to the severity of intraoperative appendicitis. For CA in children, the results of meta-analysis do not recommend the prophylactic use of AD after appendectomy. However, prospective, stratified randomized controlled trials are needed to further investigate the value of AD after appendectomy, either open or laparoscopic.

## Data Availability

The data used to support the findings of this study are available from the corresponding author upon request.
